# Resection of the primary tumour versus no resection prior to systemic therapy in patients with colon cancer and synchronous unresectable metastases (UICC stage IV): SYNCHRONOUS - a randomised controlled multicentre trial (ISRCTN30964555)

**DOI:** 10.1186/1471-2407-12-142

**Published:** 2012-04-05

**Authors:** Nuh N Rahbari, Florian Lordick, Christine Fink, Ulrich Bork, Annika Stange, Dirk Jäger, Steffen P Luntz, Stefan Englert, Inga Rossion, Moritz Koch, Markus W Büchler, Meinhard Kieser, Jürgen Weitz

**Affiliations:** 1 Department of General, Visceral and Transplant Surgery, University of Heidelberg, Heidelberg, Germany; 2 Department of Hematology and Oncology, Klinikum Braunschweig, Braunschweig, Germany; 3 Study Center of the German Surgical Society, University of Heidelberg, Heidelberg, Germany; 4 National Center for Tumor Diseases (NCT), University of Heidelberg, Heidelberg, Germany; 5 Coordination Centre for Clinical Trials (KKS), University Hospital Heidelberg, Heidelberg, Germany; 6 Institute of Medical Biometry and Informatics, University of Heidelberg, Heidelberg, Germany; 7 Department of Surgery, University of Heidelberg, Im Neuenheimer Feld 110, 69120 Heidelberg, Germany

## Abstract

**Background:**

Currently, it remains unclear, if patients with colon cancer and synchronous unresectable metastases who present without severe symptoms should undergo resection of the primary tumour prior to systemic chemotherapy. Resection of the primary tumour may be associated with significant morbidity and delays the beginning of chemotherapy. However, it may prevent local symptoms and may, moreover, prolong survival as has been demonstrated in patients with metastatic renal cell carcinoma. It is the aim of the present randomised controlled trial to evaluate the efficacy of primary tumour resection prior to systemic chemotherapy to prolong survival in patients with newly diagnosed colon cancer who are not amenable to curative therapy.

**Methods/design:**

The SYNCHRONOUS trial is a multicentre, randomised, controlled, superiority trial with a two-group parallel design. Colon cancer patients with synchronous unresectable metastases are eligible for inclusion. Exclusion criteria are primary tumour-related symptoms, inability to tolerate surgery and/or systemic chemotherapy and history of another primary cancer. Resection of the primary tumour as well as systemic chemotherapy is provided according to the standards of the participating institution. The primary endpoint is overall survival that is assessed with a minimum follow-up of 36 months. Furthermore, it is the objective of the trial to assess the safety of both treatment strategies as well as quality of life.

**Discussion:**

The SYNCHRONOUS trial is a multicentre, randomised, controlled trial to assess the efficacy and safety of primary tumour resection before beginning of systemic chemotherapy in patients with metastatic colon cancer not amenable to curative therapy.

**Trial registration:**

ISRCTN30964555

## Background

In Germany colorectal cancer represents the second most common malignancy among both sexes with a total annual incidence of 70.000 cases (50.000 patients with colon cancer) as recently reported by the Society of Epidemiological Cancer Registers in Germany (GEKID) and the Robert-Koch Institute http://www.gekid.de. At diagnosis, about 25% of colon cancer patients (i.e. 12.500 patients each year) present with distant metastases (i.e. UICC stage IV) [1]. The vast majority of these patients (i.e. 10.000 patients) remain candidates for exclusive palliative treatment [2,3]. It has been shown that some 4% of these patients present symptoms requiring urgent admission [4]. At present, the two principal treatment strategies for patients without severe tumour-related symptoms are: colonic resection followed by chemotherapy or immediate chemotherapy without prior surgery [5]. In theory, surgery delays the start of effective systemic therapy and bears the risk of severe complications and mortality. Surgery may, however, prevent development of complications caused by the primary tumour that may subsequently require emergency interventions that are associated with increased peri-operative mortality as well as less favourable long-term outcome. Given the fact that patients with metastatic disease have prolonged survival (2 years or more) with modern systemic therapy, the need for (delayed) emergency surgery may be increasing. Furthermore, removal of the primary tumour may positively affect overall survival. Although the underlying mechanisms remain unexplained at this stage, a benefit for resection of the primary tumour in stage IV disease has already been demonstrated in two randomised controlled trials (RCT) in patients suffering from metastatic renal cancer [6,7] as well as nonrandomised studies in patients with metastatic breast cancer [8,9]. The present trial is designed to evaluate whether patients benefit from surgery of the primary tumour in the setting of synchronous metastatic colon cancer not amenable to curative therapy.

### Existing evidence and need for the trial

Assuming a hazard ratio (HR) of 0.67 and 0.5, respectively, two well-designed RCTs could already demonstrate a statistically significant survival benefit for patients with metastatic renal cancer undergoing nephrectomy prior to systemic therapy. Flanigan et al. reported median overall survival times of 11.1 vs. 8.1 months (*p *= 0.01) [6] and Mickisch et al. of 17 vs. 7 months (*p *= 0.03) [7] in favour of nephrectomy.

Two recent systematic reviews detected very low quantitative as well as qualitative evidence for optimal management of asymptomatic patients diagnosed with colon cancer and synchronous metastases not amenable to curative therapy [5,10]. So far, only retrospective or prospective case series have been conducted that almost exclusively report mono-institutional experiences with small sample sizes. These studies do not allow clear conclusions, as: a) they were not hypothesis driven trials and thus lack power calculations; b) they were confounded by selection bias, as patients with favourable performance/prognostic parameters are more likely to undergo surgery; c) they included patients with cancers of both colon and rectum; d) they were mostly conducted prior to introduction of modern chemotherapeutic regimens that enable median overall survival times of 17-23 months for patients with metastatic colorectal cancer as indicated by several Phase III trials [11-13]. Against the background of these potential confounders the systematic review by Scheer et al. [10] revealed a similar benefit in median survival for patients undergoing resection of the primary tumour prior to systemic therapy in the two largest studies (Ruo et al.: 16 vs. 9 months [14]; Tebbutt et al.: 14 vs. 8.2 months [15]), whereas there was no difference in overall survival in the remaining studies. Noteworthy, the study by Tebbutt provided detailed information on complication rates showing no differences in incidence of intestinal obstruction (*p *= 0.96), peritonitis (*p *= 0.13) and gastrointestinal haemorrhage requiring hospital admission (*p *= 1.0) between both study groups [15].

### Aim of this trial

There is still considerable uncertainty, if colon cancer patients who present with incurable disease and little or no symptoms should undergo resection of the primary tumour prior to systemic therapy. A high proportion of patients undergo initial colonic resection [16]. However, this invasive procedure has a known morbidity of about 20-30% and mortality of up to 8% [17,18]. Thus, indication for colonic resection in patients without marked symptoms needs to be justified by a relevant benefit in overall survival. It is the aim of the SYNCHRONOUS trial to evaluate efficacy and safety of primary tumour resection prior to chemotherapy in patients with colon cancer and synchronous, unresectable metastases. Given the proven survival benefit for surgery in patients with metastatic renal cancer and considering the more conservative assumption applied in the RCT by Flanigan et al. [6], colonic resection may estimably prolong overall survival from 20 months in patients without surgery to 26 months. An effect of this size would represent a clinically relevant improvement and is in accordance with the retrospective studies by Tebbutt et al. and Ruo et al. [14,15]. If colonic resection has no benefit on survival, the current clinical practice can be stopped and procedure related morbidity and mortality prevented.

## Methods/design

### Trial design and randomisation

The SYNCHRNOUS trial is a randomised, controlled, multicentre, confirmatory study comparing resection of the primary tumour versus no resection prior to systemic therapy in patients with colon cancer and synchronous metastases not amenable to curative therapy.

After screening for eligibility and informed consent is obtained, patients are randomised in a 1:1 ratio into one of the following study arms:

• Resection of the primary tumour followed by systemic therapy (experimental arm)

• Systemic therapy alone (control arm)

Patients are randomised stratified for centre using a web-based, central randomisation and registration system http://www.randomizer.at.

Treatment according to randomisation, i.e. resection of the primary tumour or first cycle of systemic therapy must be carried out within 14 days after randomisation.

### Trial organisation

The SYNCHRONOUS trial will be conducted as an intergroup trial of the Study Centre of the German Surgical Society (SDGC), the German Surgical Network of Clinical Studies (CHIR-Net) together with the Colorectal Study Group of the Arbeitsgemeinschaft Internistische Onkologie (AIO) of the German Cancer Society and the Association of Certified Intestinal Centres (Arbeitsgemeinschaft Zertifizierter Darmzentren). The SDGC is responsible for the project management of the trial. Patient recruitment will take place at more than 60 trial centres throughout Europe that will be chosen out of a total of more than 100 institutions with interest in participation in the study.

### Trial population and patient recruitment

At each participating centre all consecutive patients with the new diagnosis of colon cancer and synchronous, unresectable metastases will be screened for eligibility to be enrolled in the SYNCHRONOUS trial. An interdisciplinary team at each trial centre including a surgeon and a medical oncologist/gastroenterologist determines, if patients are not amenable to curative therapy and may thus be considered for inclusion in the SYNCHRONOUS trial.

#### Inclusion criteria

• Newly diagnosed, histologically confirmed colon cancer

• Synchronous metastases not amenable to curative therapy:

Assessment by an interdisciplinary team at each trial centre including a surgeon and a medical oncologist or gastroenterologist.

• Resectable primary tumour

• ECOG performance status of 0, 1, or 2

• Patient considered to tolerate surgery and chemotherapy by the local interdisciplinary team

• ≥ 18 years of age

• Written informed consent

#### Exclusion criteria

• Rectal cancer (tumour up to 12 cm from the anal verge)

• Tumour-related symptoms or diagnostic findings requiring urgent surgery

*Definition of tumour-related symptoms*: e.g. lower gastrointestinal bleeding requiring transfusion, bowel obstruction, tumour perforation or intractable pain at site of primary tumour.

*Definition of diagnostic findings*: e.g. obstructing tumour that cannot be passed by colonoscope.

• Patients not eligible for surgery (ASA ≥ IV)

• Unequivocal extensive peritoneal metastases

• Chemo- or radiotherapy during the past 6 months

• History of another primary cancer

Exceptions: curatively treated in situ cervical cancer, curatively resected non-melanoma skin cancer or other primary solid tumour curatively treated with no known active disease present and no treatment administered for ≥ 5 years prior to randomisation.

• Expected lack of compliance

### Trial interventions

#### Experimental arm: resection of the primary tumour prior to systemic therapy

Patients in the experimental arm will undergo resection of the primary tumour prior to receiving systemic chemotherapy. Surgery has to be performed within 14 days after randomisation. Systemic therapy should be started within 8 weeks after surgery.

The type of surgical procedure depends on the location of the tumour and is thus performed as (extended) right hemicolectomy, (extended) left hemicolectomy, rectosigmoid resection or subtotal colectomy. In certain cases surgeons may perform a segmental colonic resection, if considered adequate by the executing surgeon. If possible and considered safe for the patient, resections should be performed with adequate local lymphadenectomy as it is associated with low additional morbidity [19]. However, the decision to perform a lymphadenectomy is left at the discretion of the executing surgeon and is documented in the CRF. A complete (R0) resection of the tumour should be performed. However, if a R0 resection is not considered to be safe and adequate (e.g. due to involvement of adjacent structures such as the ureter, stomach, pancreas etc.) surgeons may perform a R2 resection. The kind of resection should be documented in the case report forms (CRF).

The executing surgeon may also decide whether to perform a primary anastomosis or to create a stoma after colonic resection. Open and laparoscopic colectomy may be performed.

The principles of systemic therapy in the experimental arm are identical to those for the control arm.

#### Control arm: systemic therapy without previous resection of the primary tumour

Patients in the control arm receive primary systemic chemotherapy without previous resection of the primary tumour. Systemic therapy has to start within 14 days after randomisation. There is no predefined protocol of systemic therapy. The executing medical oncologist determines patients' treatment. Chemotherapy according to local practice and/or current guidelines is recommended (e.g. the German S3-guideline for colorectal cancer [20]). Examples for such chemotherapy regimens are (oral) fluoropyrimidin-based combination therapy with Oxaliplatin or Irinotecan such as FOLFOX-4, FOLFOX-6, FOLFIRI, CAPOX, XELOX with or without targeted therapy such as Bevacizumab or Cetuximab for K-ras wild-type tumours). However, as actually administered therapy depends on various factors (e.g. patients' comorbidities, development of toxicities) and may change over time due to emerging evidence, the administered systemic therapy is documented carefully for each patient in both study arms and is considered as a covariate in multivariate analysis (in addition to centre as factor in multivariate analysis). At each follow-up visit documentation of systemic therapy includes the administered agents (protocol), possible dose reductions and delays of planned chemotherapy cycles. Dose adjustment, duration and termination of systemic therapy are at the discretion of the executing medical oncologist. In case of progression systemic therapy may be switched to a second (and third) line regimen. The choice of the second and third line regimen is also at the discretion of the executing medical oncologist.

Patients may also discontinue systemic therapy in the following instances:

• Occurrence of adverse events, if discontinuation is desired or considered necessary by the patient and/or the medical oncologist

• Request by the patient

• Occurrence of pregnancy during treatment

• Lack of subject compliance

In case of adverse events requiring discontinuation, systemic therapy may be restarted, if considered appropriate by the executing medical oncologist.

Patients may be enrolled in further chemotherapy trials.

### Additional treatments

Owing to the palliative situation, patients in both treatment arms may receive any concomitant medications or treatments deemed necessary to provide adequate supportive care. In particular, patients may receive analgetic and antiemetic therapy for relief of symptoms directly related to the disease and/or chemotherapy.

Based on data of randomised phase III trials 6-10% of patients with colorectal cancer and unresectable metastases may become operable after treatment with modern chemotherapy regimens and subsequently undergo surgery with curative attempt [13,21]. Patients who become operable during chemotherapy may undergo treatment of the primary tumour (patients in the control arm) or metastases (patients in the experimental and control arm) with curative intent. In this scenario, patients may also undergo radiofrequency ablation (RFA) of metastatic lesions with curative attempt. The decision to initiate curative therapy has to be made by the members of the interdisciplinary team at the recruiting institution. These patients will be included in the intention-to-treat analysis.

Patients randomised to the control arm may, moreover, undergo interventional therapy for local complications (e.g. colonic stents, laser therapy). However, development of local complications will be documented as a secondary endpoint as will be the need for interventions. The decision to refer patients in the control arm for palliative tumour resection due to local complications is at the discretion of the multidisciplinary team at the recruiting institution.

The duration of treatment breaks before and after interventional procedures is at the discretion of the multidisciplinary team at the recruiting site according to the actual extent of the procedure.

### Study objectives and endpoints

The primary objective of the present trial is to investigate, whether resection of the primary tumour prolongs survival of patients with colon cancer and synchronous metastases not amenable to curative therapy. The primary hypothesis is that resection of the primary tumour prolongs survival from 20 to 26 months compared to systemic therapy without prior tumour resection.

It is the secondary objective of the trial to evaluate short- and long-term safety of both treatment strategies as well as subsequent curative procedures, the course of tumour markers and patients' quality of life.

#### Primary endpoint

The primary efficacy endpoint is *overall survival*, defined as time from randomisation date to date of death due to any reason. After randomisation patients will be followed up at intervals of three months for a minimum duration of 36 months or until death. Patients who have not died by the end of follow-up will be censored at their last contact date, as will be those patients who will be withdrawn from the study for a reason other than death (e.g. loss to follow-up).

#### Secondary endpoints

• Time-to-development of primary tumour related local symptoms (control arm):

Time from randomisation date to date of first appearance of tumour related local symptoms requiring hospitalisation and/or therapy (except for use of laxatives)

• Primary tumour complications (control arm):

Frequency and kind of local complications related to the primary tumour:

- *Lower gastrointestinal bleeding: *Evidence of lower gastrointestinal bleeding such as positive Haemoccult test or apparent blood that is not attributable to other causes (e.g. haemorrhoids), results in a drop of systemic haemoglobin and requires transfusion or interventional therapy (e.g. endoscopic control of haemorrhage)

- *Bowel obstruction: *Symptoms of ileus/subileus, i.e. abdominal pain and absence of bowel movement together with evidence of bowel obstruction from abdominal x-ray or CT scan that require hospitalisation and/or interventional therapy (e.g. stent placement, laser recanalisation, operation with bowel resection, creation of a stoma or bypass)

- *Tumour perforation*: Clinical symptoms suspicious of intestinal perforation (e.g. abdominal pain and tenderness, fever, elevated infectious parameters) together with evidence of bowel perforation by imaging (free air on abdominal x-ray, CT scan) or on laparotomy.

• Intervention due to primary tumour complication (control arm):

• Frequency and kind of operative or non-operative interventional therapy for complications of the primary tumour:

- *Operation with colonic resection*

- *Operation with creation of a stoma*

- *Operation with creation of an intestinal bypass*

- *Endoscopic placement of a stent*

- *Endoscopic recanalisation (e.g. by laser)*

- *Endoscopic control of haemorrhage (e.g. clips, submucosal injections)*

• Administration of systemic therapy (experimental and control arm):

Proportion of patients, who actually receive systemic therapy. Furthermore, the administered agents (i.e. chemotherapy protocol), dose reductions and delays of chemotherapy cycles are documented for both study arms.

• Peri-operative morbidity (experimental arm):

Frequency and kind of peri-operative complications after resection of the primary tumour until post-operative day 30 (Visit 2a). Complications are graded according to the Dindo classification (*see *Additional file 1*: Appendix A*).

- *Anastomotic leakage*

The diagnosis of anastomotic leakage after colonic resection is made considering the proposed definition of anastomotic leakage after rectal resection [22]: Defect of the intestinal wall integrity at the anastomotic site leading to a communication between the intra- and extraluminal compartments (detection by imaging or on re-laparotomy). An abscess close to the anastomosis is also considered as anastomotic leakage. The severity of an anastomotic leakage is graded as follows:

Grade A: Anastomotic leakage requiring no active therapeutic intervention.

Grade B: Anastomotic leakage requiring active therapeutic intervention but manageable without re-laparotomy.

Grade C: Anastomotic leakage requiring re-laparotomy.

- *Postoperative ileus*

Obstructive symptoms after surgery (e.g. abdominal distension, vomiting) with the need to stop food intake and/or insert a gastric tube. Radiological confirmation by plain abdominal x-ray or CT scan is required.

- *Surgical site infection (CDC-Definition, see *Additional file 1*: Appendix B)*

- *Intraabdominal Abscess*

Intraabdominal collection of purulent or infected fluid (confirmed by culture) confirmed by interventional drainage or on surgical re-intervention.

- *Postoperative haemorrhage*

Drop of systemic haemoglobin ≥ 3 g/dl compared to postoperative baseline level and/or need for transfusion of > 2 units of packed red blood cells due to intra-abdominal haemorrhage as indicated by blood loss via the abdominal drains and/or free abdominal fluid/hematoma on imaging/re-operation.

- *Deep vein thrombosis*

Clinical evidence (e.g. painful, swollen, warm, livid leg) of a deep thrombosis located in a leg or pelvic vein confirmed by duplex sonography or CT-angiography, which was not *previously known*.

- *Pulmonary embolism*

Clinical (e.g. tachycardia, dyspnoea) suspicion of pulmonary embolism confirmed by spiral computed tomography or lung perfusion scintigraphy.

- *Pulmonary infection*

At least 3 of 4 of the following: temperature > 37.5°C, purulent tracheal secretion, white blood count > 12 000 or < 4,500/ml, elevated CRP level together with radiological evidence of pulmonary infection.

- *Renal failure*

Postoperative doubling of pre-operative serum creatinine level or need for dialysis or hemofiltration (in patients who were not on dialysis pre-operatively).

- *Cerebral insult*

Clinical symptoms suspicious of an (ischemic or non-ischemic) cerebrovascular event with confirmation by CT or MRT.

- *Myocardial infarction*

Electrocardiogram (NSTEMI or STEMI) and enzyme (Troponin I) changes suggestive of myocardial infarction or evidence of myocardial infarction on coronary angiogram.

- *Hospital stay*

The length of hospital stay registered from the first day after the operation until the day of discharge.

• Peri-operative mortality (experimental arm):

30-day mortality or in-hospital mortality during initial hospital stay for resection of the primary tumour (i.e. deaths occurring after patients' discharge from the hospital but within 30 days after resection of the primary tumour are documented as peri-operative mortality).

• Interventions with curative intent (experimental and control arm):

Frequency and kind of interventions performed with curative intent:

- Potentially curative resection of the primary tumour and metastases (control arm)

- Potentially curative resection of metastases (experimental arm).

- Radiofrequency ablation (RFA) of metastases

The decision for an intervention with curative intent is made by the members of the interdisciplinary team at the participating institution.

• Course of tumour markers:

The course of the tumour markers carcinoembryonic antigen (CEA) and carbohydrate antigen (CA) 19-9 will be monitored in both study arms during the course of therapy. It is a secondary objective to evaluate the influence of primary tumour resection on the circulating levels of these tumour markers.

• Quality of Life:

Quality of life is measured using the EQ-5D™ questionnaire of the EuroQol Group. In addition, the EORTC QLQ-C30 instrument and the specific colorectal module CR29 is used.

### Trial implementation

There are 14 pre-specified study visits (V1 - V14) within the SYNCHRONOUS trial. In V1 patients will be screened according to the eligibility criteria and asked for written informed consent. Within V1 participating patients are also asked to complete the first quality-of-life form.

After enrolment into the study patients will be allocated randomly to either study arm (V2). The protocol requires that the actual start of therapy (i.e. resection of the primary tumour or first cycle of systemic therapy) is carried out within 2 weeks after randomisation to ensure comparable follow-up periods within and between both study arms. An additional visit (Visit 2a) will be performed in patients allocated to the experimental arm to assess early postoperative outcomes until post-operative day 30 (± 7 days). After randomisation patients will be followed-up every 3 months (± 7 days) in both study arms. The frequency and scope of study visits are in line with routine clinical care of patients with metastatic colon cancer. In these study visits (V3 - V14) data on the primary endpoint and secondary endpoints are documented including data on safety, complications, administered therapies (chemotherapy protocols, interventional therapies and surgical procedures) and tumour markers. In addition, quality of life will be assessed on V3 (3 months after V2), V4 (6 months after V2) and then every 6 months (i.e. on V6, V8, V10, V12, V14). V14 is the end-of-study visit, which will take place at a maximum of 36 months follow-up after V2. Peri-operative laboratory tests and those during chemotherapy cycles will be performed at discretion of the executing investigator based on local standards. Table 1 summarises the intended frequency and scope of study visits.

**Table 1:** Investigation scheme in the SYNCHRONOUS trial

Documentation	Visit 1	Visit 2	**Visit 2a**^1)^	Visit 3 - 13	Visit 14
	**Screening**	**Maximum 2 weeks before start of therapy**	**30 days after surgery**	**q 3 months after V2**	**36 months after V2**

Eligibility criteria	**X**				

Baseline data, demographics	**X**				

Laboratory analyses	**X**				

Tumor markers	**X**			**X**	**X**

Randomization		**X**			

Administered therapies			**X**	**X**	**X**

Chronic use of analgetic medication	**X**			**X**	**X**

Assessment of com-plications & safety			**X**	**X**	**X**

Primary endpoint			**X**	**X**	**X**

Secondary endpoints			X	**X**	**X**

Quality of life	**X**			**X**^2)^	**X**

^1) ^Visit 2a will be performed in patients allocated to the experimental arm only. ^2) ^Quality of life will be assessed on V3 (3 months after V2), V4, (6 months) and then every 6 months

### Sample size

Based on the results obtained from a literature search, a median overall survival time of 20 months is expected for the control group (Fuchs et al. [12], Saltz et al. [13], Van Cutsem et al. [23], Souglakos et al. [21], Seymour et al. [11]). An improvement to 26 months by the surgical intervention is considered to be clinically relevant and achievable (Flanigan et al. [6], Ruo et al. [14], Tebbutt et al. [15]). For exponentially distributed survival times, this treatment group difference corresponds to a hazard ratio of 1.3. The accrual period amounts to 24 months and the follow-up period to 36 months. To detect a hazard ratio of 1.3 with a two-sided test for treatment effect at a significance level of 5% within a Cox model without covariates with a power of 85%, 694 patients (347 per group) have to be included in the analysis, leading to a total number of events of 522. These calculations are based on the formula for the log-rank test [24] and were performed with ADDPLAN 5.0. Due to the asymptotic equivalence of the test statistic of the Cox model without covariates and the log-rank test statistics under the assumed model, the sample size calculation holds also true for the unadjusted Cox model. It can be expected that inclusion of the covariates centre, age, and administered systemic therapy in the analysis will further increase the power as compared to the above calculations that are based on the Cox model without covariates.

Based on the current knowledge about the treatments under investigation, it could not be ruled out that the experimental intervention may show no effect or even a negative effect in the early part of the follow-up as compared to the control group, and that its advantage becomes clear later on. To assess the robustness of the study power, simulations were performed for various parameter scenarios reflecting this situation. A linear increase of the mortality in the control group and an instantaneous increase in the experimental group by rates between 5% and 10% during the first three months of the follow-up were considered. Crossing of the survival curves was assumed to occur after three months and exponential survival thereafter leading overall to the same median survival times as specified above. For the sample size of 694 patients, the resulting power for the two-sided test for treatment effect within the Cox model without covariates varied between 84.5% and 90.3% thus demonstrating robustness of the study power to the assumptions made with respect to the course of the survival curves (simulations performed in SAS, version 9.1; 100,000 replications were performed for each parameter scenario). We repeated these simulations, but now applying the robust test proposed by Lin and Wei [25] which will be applied in the analysis. For the robust test, power values in the range from 84.6% to 90.5% were obtained. Furthermore, considering the same scenarios we performed simulations under the null hypothesis. The estimated type I error rate for a nominal two-sided significance level of 5% were between 4.95% and 5.08% for the standard Cox model and between 4.78% and 4.98% for the robust approach. It can be concluded that the robust test leads to virtually the same power in the realistic scenarios we considered in our simulations while at the same time assuring appropriate type I error rate even if the proportional hazards assumption is violated.

The loss-to-follow up rate can be assumed to be very low (less than 1% in Van Cutsem et al. [23]). However, it is expected that in the experimental group a number of patients will not receive standard systemic treatment after resection of the primary colon tumour (about 20%; see Ruo et al. [14]). Furthermore, some patients will stop standard systemic treatment early due to lack of tolerability (about 20%; see Saltz et al. [13]). Although these patients will be included in the intention-to-treat analysis, there might be some dilution of the treatment effect due to these protocol violations. This is accounted for by randomising further 15% of the calculated number of patients, i.e., a total of 800 patients (400 per group). It is assumed that further 100 patients will have to be screened to achieve this number of randomised patients.

### Statistical analysis

#### Confirmatory analysis

The confirmatory analysis of the primary efficacy endpoint will be conducted according to the intention-to-treat principle, i.e., all randomised patients will be included and will be analysed in the treatment group where they were allocated to by randomisation. The test for treatment group difference will be performed at the two-sided type I error rate 5% within a Cox proportional hazards model that takes into account the covariates centre, age, and administered systemic therapy. The robust sandwich estimate of the covariance matrix proposed by Lin and Wei [25] is used for the test for treatment effect. As shown in [25], this test allows valid statistical inference also in situations where the proportional hazards assumption is violated, which may be the case in the current trial. Drop-out and lost-to-follow-up as well as non-occurrence of death within the follow-up period are treated as censoring events. Overall survival will be displayed for each intervention group based on the Kaplan-Meier estimates as well as on the estimates obtained from the Cox regression model, and the corresponding two-sided 95% confidence interval for the hazard ratio will be calculated.

No interim analysis is planned for this trial. As the follow-up for the primary endpoint is 36 months, and the planned recruitment time 24 months, the results of an interim analysis would arise after completion of the recruitment and treatment period. Accordingly, an interim analysis could not cause a reduction in sample size and was therefore not implemented.

#### Further analyses

Descriptive methods will be used for the analysis of the secondary outcomes. Time-to-event endpoints will be analysed as described for the primary endpoint. Binary secondary endpoints will be analysed using logistic regression models. Appropriate summary measures of the empirical distributions as well as descriptive p-values will be calculated. Graphical methods will be applied to visualise the findings of the study. Additionally, sensitivity analyses will be conducted for different populations (per protocol population of those patients that show no relevant protocol violations, appropriate subgroups). The safety analysis will be based on all randomised patients who were treated with any of the interventions under investigation. The analysis will include calculation and comparison of the rates of complications and serious adverse events as well as graphical display of the time-course. All analyses will be done using SAS version 9.1 or higher.

### Data management and quality assurance

The investigator or a designated representative must enter all protocol-required information in the electronic case report form (eCRF). Any entry and correction in the Remote Data Entry System will be documented automatically in an audit file. Once the documentation of a patient is completed and checked for plausibility the investigator is asked to date and sign it via electronic identification. Documentation of quality of life questionnaires will be done on paper based questionnaires.

#### Control of data consistency

Automatic checks for data completeness, validity and plausibility will be programmed by the data management group of the Institute of Medical Biometry and Informatics (IMBI), University of Heidelberg, and queries will be generated. The investigator or the designated representatives are obliged to clarify or explain the queries. If no further corrections are to be made in the database, it will be closed and used for statistical analysis. All data management procedures will be carried out according to the current Standard Operating Procedures (SOPs) of the IMBI.

#### Quality control and monitoring

Clinical monitoring will be performed by the Coordination Centre for Clinical Trials (KKS) Heidelberg, an institution which is independent from other trial staff. Monitoring procedures will be adapted to the study specific risk for the patients. Interpretation of standard operating procedures (SOP) of the KKS to ensure patients' safety and integrity of the clinical data, e.g. primary endpoint in adherence to study protocol. Pre-study visits will be performed in centres interested to participate in the study, to ensure high compliance quality of the participating centres concerning e.g. patient recruitment and documentation.

External monitoring of entries in the electronic CRF will be done by independent monitors from KKS Heidelberg. Regular on-site monitoring visits are planned at all sites depending on the recruitment rate and quality of the data. Monitoring strategy and extent of source data verification (SDV) are described in a trial specific monitoring manual.

### Ethical and legal considerations

The SYNCHRONOUS trial is conducted in line with either the Declaration of Helsinki (Tokyo, Venice, Hong Kong, Somerset West and Edinburgh amendments) or the laws and regulations of the country, whichever provides the greatest protection of the patient.

The protocol has been written, and the study will be conducted according to the ICH Harmonized Tripartite Guideline for Good Clinical Practice http://www.ifpma.org/pdfifpma/e6.pdf.

The trial protocol, patient information and informed consent sheets have been approved by the independent ethics committee of the University of Heidelberg, Medical School (S-073/2011) and by the competent ethics committees of all participating trial centres. The SYNCHRONOUS trial has been registered at the ISRCTN database (ISRCTN30964555; http://www.isrctn.org).

All patients will be informed of the aims of the study, the possible adverse events, the procedures and possible hazards to which he/she will be exposed, and the mechanism of treatment allocation. Furthermore, it is the responsibility of the investigator to explain patients their duties within the trial. They will be informed as to the strict confidentiality of their patient data, but that their medical records may be reviewed for trial purposes by authorised individuals other than their treating physician. An example of a patient informed consent statement is given as an appendix to this protocol.

During the trial, patients will be identified solely by means of their year of birth and individual identification code (screening number, randomisation number). Trial findings will be stored in accordance with local data protection law/ICH GCP-Guidelines and will be handled in strictest confidence. For protection of these data, organisational procedures are implemented to prevent distribution of data to unauthorised people.

### Trial sponsorship and funding

The SYNCHRONOUS trial is sponsored by the Universitätsklinikum Heidelberg, represented by the Commercial Director. The trial is funded exclusively by the Deutsche Forschungsgemeinschaft (WE 3548/5-1).

### Current status

The trial protocol has been approved by the independent ethics committee of the University of Heidelberg, Medical School. At the time of manuscript preparation applications have been sent to the majority of the affiliated local ethics committees of the participating institutions.

Prior to the start of the study, all participating centres were trained and introduced into all study specific procedures. For this purpose an investigator's meeting was held in Heidelberg in October 2011. During this two-day workshop investigators and study nurses from the participating institutions were instructed in the general principles of clinical trials (day one) and the study specific implementation of the SYNCHRONOUS trial (day two). The commitment of all participating institutions to include patients in this important trial was strengthened and the participants used the opportunity to discuss protocol specific subjects and exchanged their experience from previous randomized controlled trials. As recruitment of patients was considered a critical point of the study, the participants expressed their strong will to ensure sufficient recruitment of patients and discussed strategies to optimize patient recruitment.

## Discussion

The SYNCHRONOUS trial is designed to evaluate efficacy and safety of primary tumour resection prior to systemic chemotherapy in patients with newly diagnosed CRC and synchronous, unresectable metastases. Even though enormous progress has been made in the treatment of patients with CRC within the past three decades [26], the optimal management of patients with metastatic disease not amenable to curative therapy who present without severe primary tumour related symptoms has remained controversial. While randomised controlled trials on patients with renal cell carcinoma showed a survival benefit by removal of the primary tumour [6,7], there has been a lack of well-designed and controlled trials on the prognostic value of primary tumour resection in CRC as indicated by two systematic reviews [5,10].

In the SYNCHRONOUS trial the two strategies of primary tumour resection prior to chemotherapy and immediate chemotherapy without previous resection of the primary tumour are compared in a prospective, randomised fashion. One should note that within this trial two therapeutic strategies are compared rather than two specific therapies with the aim to increase external validity and ensure feasibility. For this reason there is no pre-specified chemotherapy regimen to be applied within the study arms of the trial and the choice for the chemotherapy protocol is at discretion of the medical oncologist in charge at each participating institution considering current guidelines. However, the applied chemotherapy in both study arms is documented accurately and will be included as a covariate in the multivariate analysis. Similarly, the study protocol does not call for specific surgical procedures for primary tumour resection that should rather be performed in line with the standards of the participating institution, the patient's condition, the location of the tumour and the surgeon's preference. Colonic resection may therefore be performed laparoscopically or via laparotomy. Furthermore, the executing surgeon may decide whether or not to create a primary anastomosis. As in selected patients therapy with modern chemotherapy protocols may cause marked tumour regression that may enable further therapeutic action with curative intent, colonic resection with adequate lymphadenectomy is recommended, though this is not mandatory requirement and the decision to perform lymphadenectomy remains at the discretion of the executing surgeon. However, patients who become candidates for curative resection after responding to chemotherapy may receive further treatment with curative intent. Additional treatments will be documented and these patients will be analysed within the intention-to-treat population.

## Conclusion

The SYNCHRONOUS trial is a multicentre, randomised controlled trial to evaluate efficacy and safety of primary tumour resection in patients with metastatic colon cancer who do not suffer from local symptoms of the primary tumour. Its results may help to optimise the management of colon cancer patients who are not amenable to curative therapy.

## Competing interests

The authors declare that they have no competing interests.

## Authors' contributions

This study was designed by NNR, FL and JW. The article was written by NNR. MeK and SE performed the sample size calculation and planned the statistical analyses. CF, UB, SPL, IR, MK and MWB are involved in trial implementation and critically revised the manuscript. All authors have read and approved the manuscript.

## Funding

This trial is funded by the Deutsche Forschungsgemeinschaft (WE 3548/5-1)

## Pre-publication history

The pre-publication history for this paper can be accessed here:

http://www.biomedcentral.com/1471-2407/12/142/prepub

## Supplementary Material

Additional file 1 **Classification of perioperative complications**. Click here for file
