# Supplementary material for: Resection of the primary tumour versus no resection prior to systemic therapy in patients with colon cancer and synchronous unresectable metastases (UICC stage IV): SYNCHRONOUS - a randomised controlled multicentre trial (ISRCTN30964555)
Source: BMC Cancer. 2012 Apr 5;12:142. doi: 10.1186/1471-2407-12-142 (PMC3348093; doi:10.1186/1471-2407-12-142)
Supplement: Additional file 1 — Classification of perioperative complications. [file 1471-2407-12-142-S1.PDF]

Appendix A:

Classification of perioperative complications

Grade	Definition
Grade I	Any deviation from the normal postoperative course without the need for pharmacological treatment or surgical, endoscopic, and radiological interventions
	Allowed therapeutic regimens are: drugs as antiemetics, antipyretics, analgetics, diuretics, electrolytes, and physiotherapy. This grade also includes wound infections opened at the bedside
Grade II	Requiring pharmacological treatment with drugs other than such allowed for grade I complications
	Blood transfusions and total parenteral nutrition are also included
Grade III	Requiring surgical, endoscopic or radiological intervention
Grade IIIa	Intervention not under general anesthesia
Grade IIIb	Intervention under general anesthesia
Grade IV	Life-threatening complication (including CNS complications)* requiring IC/ICU management
Grade IVa	Single organ dysfunction (including dialysis)
Grade IVb	Multiorgan dysfunction
Grade V	Death of a patient
Suffix "d"	If the patient suffers from a complication at the time of discharge (see examples in Table 2), the suffix "d" (for "disability") is added to the respective grade of complication. This label indicates the need for a follow-up to fully evaluate the complication.

*Brain hemorrhage, ischemic stroke, subarrachnoidal bleeding, but excluding transient ischemic attacks.
CNS, central nervous system; IC, intermediate care; ICU, intensive care unit.

APPENDIX B:

**Summary of the definitions of superficial and deep surgical site infections according to the Centers for Disease Control (CDC)**	
**Superficial incisional SSI**	**Deep incisional SSI**
Superficial incisional SSI must meet the following two criteria :<br><br>• occur within 30 days of procedure<br>• involve only the skin or subcutaneous tissue around the incision.<br><br>*plus*<br><br>At least one of the following criteria:<br><br>• purulent drainage from the incision<br>• organisms isolated from an aseptically obtained culture of fluid or tissue from the incision<br>• at least one of the following signs or symptoms of infection - pain or tenderness, localised swelling, redness or heat - and the incision is deliberately opened by a surgeon, unless the culture is negative<br>• diagnosis of superficial incisional SSI by a surgeon or attending physician<br><br>The following are not considered superficial SSIs:<br><br>• stitch abscesses (minimal inflammation and discharge confined to the points of suture penetration)<br>• infection of an episiotomy or neonatal circumcision site<br>• infected burn wounds<br>• incisional SSIs that extend into the fascial and muscle layers *(see* deep SSIs).	Deep incisional SSI must meet the following three criteria :<br><br>• occur within 30 days of procedure (or one year in the case of implants)<br>• are related to the procedure<br>• involve deep soft tissues, such as the fascia and muscles.<br><br>*plus*<br><br>At least one of the following criteria:<br><br>• purulent drainage from the incision but not from the organ/space of the surgical site<br>• a deep incision spontaneously dehisces or is deliberately opened by a surgeon when the patient has at least one of the following signs or symptoms - fever (>38℃), localised pain or tenderness - unless the culture is negative<br>• an abscess or other evidence of infection involving the incision is found on direct examination or by histopathologic or radiological examination<br>• diagnosis of a deep incisional SSI by a surgeon or attending physician.<br><br>Infection that involves both superficial and deep incision sites is classified as deep incisional SSI !
